# Ocular Findings and Visual Function in Children Examined during the Zika Health Brigade in the US Virgin Islands, March 2018

**DOI:** 10.3390/tropicalmed6020066

**Published:** 2021-04-29

**Authors:** S. Grace Prakalapakorn, Lucas Bonafede, Linda Lawrence, Daniel Lattin, Nicola Kim, Richard D. House, Braeanna Hillman, Leah de Wilde, Cosme Harrison, Nicole Fehrenbach, Shana Godfred-Cato, Megan R. Reynolds, Esther M. Ellis

**Affiliations:** 1 Department of Ophthalmology, Duke University, Durham, NC 27710, USA; lucasbonafede@gmail.com (L.B.); daniel.lattin@nemours.org (D.L.); nicola.kim@duke.edu (N.K.); 2 Private Practice Ophthalmology, Salina, KS 67401, USA; lmlawrencemd@gmail.com (L.L.); rickydhouse@gmail.com (R.D.H.); 3 Chickasaw Health Consulting LLC, Norman, OK 73071, USA; bmhillman@gmail.com (B.H.); Leah.Dewilde@doh.vi.gov (L.d.W.); Cosme.Harrison@doh.vi.gov (C.H.); 4 United States Virgin Islands Department of Health, Christiansted, VI 00820, USA; esther.ellis@doh.vi.gov; 5 United States Virgin Islands Department of Health, Charlotte Amalie, VI 00802, USA; 6 Centers for Disease Control and Prevention, National Center on Birth Defects and Developmental Disabilities, Division of Birth Defects and Infant Disorders, Atlanta, GA 30341, USA; ekk5@cdc.gov (N.F.); nzt6@cdc.gov (S.G.-C.); xah6@cdc.gov (M.R.R.)

**Keywords:** congenital Zika infection, ocular findings, visual function, visual impairment, vision screening

## Abstract

Among children born with laboratory-confirmed Zika virus (ZIKV) infection, visual impairment (VI) can occur despite normal ocular structure. The objective of this report is to describe ocular findings and visual function among children examined during the Department of Health Zika Health Brigade (ZHB) in the United States Virgin Islands in March 2018. This analysis is based on a retrospective chart review of children eligible to participate in the ZHB (i.e., part of the US Zika Pregnancy and Infant Registry) and who were examined by ophthalmologists. Eighty-eight children attended the ZHB. This report includes 81 children [48 (59.3%) males] whose charts were located [average gestational age = 37.6 weeks (range: 27.6–41.3) and average adjusted age at examination = 9.1 months (range: 0.9–21.9)]. Of those examined, 5/81 (6.2%) had microcephaly at birth, 2/81 (2.5%) had a structural eye abnormality, and 19/72 (26.4%) had VI. Among children with normal ocular structure and neurologic examination, 13/51 (25.5%) had VI. Despite a low incidence of abnormal ocular structure and microcephaly, about a quarter of children examined had VI. Our findings emphasize that ophthalmological examinations should be performed in all children with suspicion for antenatal ZIKV infection, even children with normal ocular structure and neurologic examination.

## 1. Introduction

First isolated in Uganda in 1947, Zika virus (ZIKV), a single-stranded ribonucleic acid flavivirus, is primarily transmitted by the *Aedes* mosquito, but can also be transmitted sexually, intrauterine (mother-to-fetus during pregnancy), and perinatally (mother-to-infant at delivery) [1,2]. While most people infected with ZIKV are asymptomatic or only have mild symptoms of fever, maculopapular rash, conjunctivitis, or arthralgias, in 2013–2014 ZIKV was first noted to be associated with Guillain-Barre syndrome [1]. In 2017, it was reported that infection with ZIKV during pregnancy can cause a recognizable pattern of structural anomalies and functional disabilities known as Congenital Zika syndrome (CZS) [3]. The five features unique to CZS or rarely seen with other congenital infections include: (1) Severe microcephaly with partially collapsed skull; (2) Thin cerebral cortices with subcortical calcifications; (3) Macular scarring and focal pigmentary retinal mottling; (4) Congenital limb contractures; and (5) Marked early hypertonia and symptoms of extrapyramidal involvement [3]. The most recent ZIKV outbreak identified in the Americas in 2015 has had a wide geographic distribution, being reported in 86 countries and territories [4]. 

Most of our knowledge on ocular findings and visual impairment have come from South America, in particular Brazil [5,6,7,8,9,10,11,12]. The most commonly reported ocular findings associated with congenital ZIKV infection include macular (i.e., chorioretinal scarring and focal pigmentary changes) and optic nerve abnormalities (i.e., hypoplasia, cupping, and atrophy) [5,6,7,8,13]. In cases of presumed and confirmed ZIKV infection both with and without structural ocular abnormalities, studies have reported abnormal visual acuity and function in children [5,6,7,8,11,12]. Among children born with laboratory-confirmed ZIKV infection, severe visual impairment can occur despite normal ocular structure due to presumed cortical visual impairment [5,6,7,8].

As far as we are aware, there have been no studies published on the ocular findings and visual function in a cohort of infants in the United States (US) or US territories. The US Zika Pregnancy and Infant Registry (USZPIR) is a surveillance system collecting information about pregnancy and infant/child outcomes among pregnancies with laboratory evidence of confirmed or possible ZIKV infection [14,15]. A Zika Health Brigade (ZHB) was carried out in the US Virgin Islands (USVI) in March 2018, which included evaluation of infants born to mothers with possible or confirmed infection with ZIKV during pregnancy [16]. During the ZHB, infants were evaluated by pediatricians and pediatric neurologists, ophthalmologists, and audiologists [16].

The objective of this report is to describe ocular findings and visual function of a group of children born to mothers with possible or confirmed ZIKV infection during pregnancy who were examined during the Department of Health ZHB in the USVI in March 2018.

## 2. Materials and Methods

This report was approved by the Duke University Health System Institutional Review Board, who waived the requirement for informed consent, and complied with the regulations of the US Health Insurance Portability and Accountability Act of 1996. 

Through this retrospective chart review, we only included children who met USZPIR eligibility criteria, were examined during the ZHB by ophthalmologists, and had complete charts from the ZHB available for review. USZPIR eligibility criteria included children born between 1 December 2015 and 31 March 2018 in the USVI, to mothers with confirmed or possible recent ZIKV infection as defined by (1) ZIKV infection detected by Zika ribonucleic acid nucleic acid amplification test (NAAT) on any maternal, placental, fetal, or infant specimen or (2) ZIKV or flavivirus infection detected by serologic tests of maternal, fetal, or infant specimen [14]. Confirmed ZIKV infection was defined as either: (1) positive NAAT or 2) positive or equivocal Zika Immunoglobulin M testing and ZIKV plaque reduction neutralization test (PRNT) ≥ 10 and dengue PRNT < 10. A ZHB was carried out in the USVI from 19 to 24 March 2018, which included evaluations by developmental pediatricians and pediatric ophthalmologists, neurologists, and audiologists. For this report, developmental abnormalities were considered present if the child was either categorized during the ZHB as “at risk for developmental delay” based on results of screening using the Ages and Stages Questionnaire −3 or found to have “other neurodevelopmental abnormalities” (i.e., abnormal tone, abnormal movement, arthrogryposis, or seizures) based on physical examination and medical record review by a pediatric neurologist.

**Figure 1:**
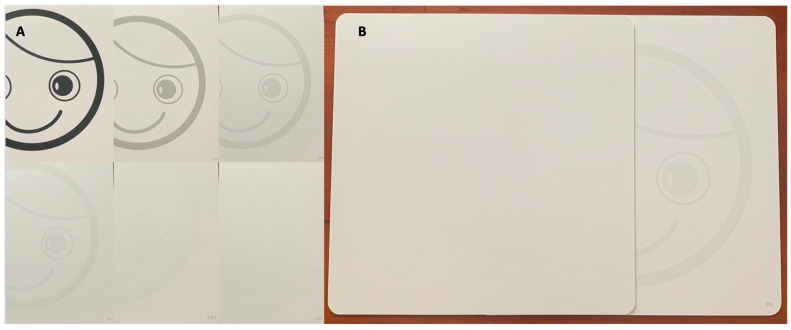
Hiding Heidi Contrast test (Good-Lite, Elgin, IL, USA). (**A**) The cards from highest (100%, top left) to lowest (1.25%, bottom right) contrast, where the % contrast can be seen at the bottom right hand corner of each card. (**B**) During testing, the Hiding Heidi Contrast card would be revealed under a white card and the examiner would look for shift of gaze or head turn toward the visual stimuli, here the card with 5% contrast is shown.

All children who presented during the ZHB had a comprehensive ophthalmologic examination performed by an ophthalmologist that consisted of evaluation of visual function, visual development, ocular motility and alignment, anterior segment examination, intraocular pressure, cycloplegic refraction, and dilated fundus examination. Visual function testing included Hiding Heidi Contrast testing (Good-Lite, Elgin, IL, USA, Figure 1), shift of gaze testing, confrontational visual fields, and accommodation. Confrontational visual fields evaluated a child’s ability to recognize a stimulus in each quadrant. Accommodation was considered abnormal if dynamic retinoscopy was abnormal. Visual development was considered abnormal if the child was unable to meet visual milestones according to adjusted age (Table 1) [17,18]. Visual impairment was considered present if the 5% or lower Hiding Heidi contrast card (Figure 1B) did not elicit a response at 30 cm, or if shift of gaze testing, confrontational visual field testing, accommodation testing, or visual development was abnormal. Intraocular pressure was checked with rebound tonometry (iCare^®^ TAO1i, iCare, Helsinki, Finland).

Statistical analysis was performed with Microsoft Excel (v2016, Microsoft Corp, Redmond, WA, USA) and JMP Pro (v15.0.0, SAS Institute Inc., Cary, NC, USA). Visual impairment results were stratified by gestational age and other clinical characteristics to evaluate for differences in occurrence of visual impairment based upon these characteristics. Due to small cell sizes, statistical significance was assessed using the Fisher’s exact test.

## 3. Results

Of 242 completed pregnancies meeting USZPIR eligibility criteria in the USVI, 88 children attended the ZHB, and complete charts were located for 81 (33.5%). Of the 81 children included in this report, 48 (59.3%) were male, mean gestational age = 37.6 weeks (range: 27.6–41.3), and mean birth weight = 2965 g (range: 755–3960) (Table 2). The adjusted age on the date of examination during the ZHB was 9.1 months (range: 0.9–21.9). Fourteen (17.3%) children had confirmed ZIKV infection and 5 (6.2%) had microcephaly at birth. Of 14 infants with confirmed ZIKV infection, 1 (7.1%) had microcephaly. Developmental abnormalities were present in 19/81 (23.5%) children evaluated where 18 (22.2%) were “at risk for developmental delay” and 5 (6.2%) had other neurodevelopmental abnormalities (Table 2). 

On ophthalmologic assessment during the ZHB, 2/81 (2.5%) children had a structural ocular abnormality, one had microphthalmia and one had optic nerve cupping. No children had a retinal abnormality. One child had abnormal ocular motility (1/80, 1.3%), one had strabismus (1/78, 1.3%), and none had nystagmus. Intraocular pressure was not elevated in any eyes tested (n = 158, range: 4–21 mmHg). Average spherical equivalent on cycloplegic refraction was +0.73 diopters (D) (standard deviation (SD) = 1.37) in right and +0.75 D (SD = 1.38) in left eyes (r = 0.99). 

Overall, visual impairment was noted in 19 (26.4%) of 72 children who completed all components of the visual function and visual development testing. The adjusted age on the date of examination of these 19 children was 7.6 months (SD = 4.9; range: 1.7–16.7). Children qualified as having visual impairment due to abnormal contrast testing in 18/75 (24.0%), abnormal shift of gaze in 6/77 (7.8%), abnormal confrontational visual fields in 1/73 (1.4%), abnormal accommodative reflex in 1/71 (1.4%), and abnormal visual milestones in 2/78 (2.6%) (Table 3). Among children who completed all components of the visual function and visual development testing, visual impairment was noted among 6 (31.6%) of 19 children born < 37 weeks gestational age, neither (0%) of the 2 children with a structural eye abnormality, 1 (20.0%) of 5 children with microcephaly, 5 (31.3%) of 16 children at risk for developmental delay, 2 (50.0%) of 4 children with other neurodevelopmental abnormalities, 5 (38.5%) of 13 children with confirmed ZIKV infection, and 13 (25.5%) of 51 children with normal eye, neurologic, and developmental examination (Table 3).

The proportion of children with visual impairment in those born preterm versus full-term and with versus without structural eye anomalies, microcephaly, developmental abnormalities, and/or confirmed ZIKV infection were not statistically significant between groups (Table 4). Among children with normal examinations without evidence of microcephaly, structural eye abnormalities, or neurodevelopmental abnormalities, 13/51 (25.5%) had visual impairment (Table 3). Among children with visual impairment, 13/19 (68.4%) had normal examinations without evidence of microcephaly, structural eye abnormalities, or neurodevelopmental abnormalities. 

## 4. Discussion

Among children born in the USVI to women who met USZPIR eligibility criteria of confirmed or possible recent ZIKV infection during pregnancy, were examined during the ZHB, and had complete charts available for review, 2.5% had structural ocular abnormalities and 26% had visual impairment. 

Among children exposed to ZIKV infection antenatally, studies have reported a broad array and prevalence of ophthalmic findings (e.g., structural ocular anomalies, glaucoma, strabismus, nystagmus, visual impairment). Six studies reporting on visual impairment in children affected by congenital ZIKV infection have all been published from Brazil (Table 5) [5,6,7,8,11,12]. One study evaluating 32 infants (mean age = 5.7 months) with neurologic or neuro-radiologic abnormalities with confirmed ZIKV infection (100% had microcephaly) found a structural ophthalmic abnormality (retinal and/or optic nerve abnormality) in 44% of children and visual impairment in 100% of eyes, due mainly to not achieving visual milestones (97%), followed by abnormal Hiding Heidi testing (65%) [6]. A later study by the same authors evaluating 119 infants (mean age = 8.5 months) with confirmed ZIKV infection found a high prevalence of microcephaly (89%), structural ophthalmic abnormalities (retinal abnormalities in 32%, optic nerve abnormalities in 27%), and visual impairment (90%), again due mainly to not achieving visual milestones (93%) followed by abnormal Hiding Heidi testing (81%) [5]. Both previous studies noted that among infants with a normal structural eye examination, ≥85% of subjects had abnormal vision testing, thought to be secondary to cortical visual impairment as ≥89% of their study population had microcephaly [5,6]. In another study, among 70 infants with microcephaly due to presumed CZS, 26% had a structural ophthalmic abnormality (retinal and/or optic nerve abnormality). Vision testing was performed in 11 children, and all (100%) were abnormal, of which, 3 (27%) had no ophthalmic structural abnormality [7]. An additional study evaluating 173 infants (3–6 months of age) with suspected ZIKV infection found 36% had microcephaly, 2% an anterior segment abnormality, 26% a retinal and/or optic nerve abnormality, and 30% abnormal visual function based on inability to fix and follow by 3–6 months of age [8]. These studies all reported a higher prevalence of structural, neurologic, and visual abnormalities in infants affected by congenital ZIKV infection than in our study. More recently, another group initially evaluated 47 infants (mean age = 6.5 months) who were exposed to or infected by ZIKV antenatally and found 9% had microcephaly, 2% a retinal abnormality, and 11% with decreased vision by teller acuity testing (50% of those with microcephaly, 100% of those with a retinal abnormality, 7% of infants without microcephaly and/or retinal abnormalities, and 0% of infants exposed to ZIKV without confirmation of infection) [11]. Then, among a subset of 75% (33/44) of these infants, including 12% with microcephaly and 3% with a retinal abnormality, they reported that over time, all children had normal visual development, except for 3% with decreased visual acuity (i.e., one child with both microcephaly and chorioretinal scarring) [12]. In our study, 6% had microcephaly at birth, 2.5% a structural ophthalmic abnormality, and 26% visual impairment, 95% due to abnormal Hiding Heidi testing. Stratifying visual impairment by gestational age and other clinical characteristics to evaluate for differences in occurrence based upon these characteristics we found that the percentage of those with visual impairment was noted to be highest among those with other neurodevelopmental abnormalities (50%); followed by those with confirmed ZIKV infection (38.5%); those born preterm (31.6%); those at risk for developmental delay (31.3%); those with a normal eye, neurologic, and developmental examination (25.5%); those with microcephaly (20.0%); and neither (0%) of the children with a structural eye abnormality was noted to have visual impairment. Despite these variances, we did not find a statistically significant difference in the proportion of children with visual impairment among those born preterm versus full-term and among those with versus without structural eye anomalies, microcephaly, developmental abnormalities, and/or confirmed ZIKV infection.

We believe the differences in numbers between previous studies and our report is mainly due to differences in our inclusion criteria. Similar to the more recent publication which included infants exposed to (but not confirmed to be infected by) ZIKV, we included children at the more mild spectrum of disease, whereas the earlier studies included children more severely affected by ZIKV infection [5,6,7,8], e.g., one study included only those with microcephaly [7]. Unfortunately, the findings in the more recently published studies are not directly comparable to ours as visual function was not measured using the same metrics. In our study even among the children with microcephaly, only 20% had visual impairment, versus 71–100% in earlier studies [6,7,8]. Additionally, the average age of those examined in our report was older versus other studies [5,6,7,8]. Despite these differences, our broader inclusion criteria are a strength of this report, allowing us to report the occurrence of these findings within a population with a wider spectrum of disease (i.e., all cases of laboratory-suspected ZIKV infections regardless of presence of Zika-associated birth defects or neurodevelopmental abnormality).

Consistent with previous studies, we found that visual impairment can be present in the setting of normal ocular structure [5,6,7,8]. Our study had a relatively low incidence of structural ocular abnormalities, strabismus, or nystagmus, and among infants with a normal ocular structure and neurodevelopmental examinations, 26% had visual impairment. Most infants in our cohort (and previous studies) [5,6] were categorized as visually impaired based on decreased contrast sensitivity testing (i.e., Hiding Heidi testing). Contrast sensitivity is a recommended part of a functional visual assessment [19] and is felt to give useful information regarding the potential for a child to become a visual reader and predict and explain their present and future performance [20]. While age-related norms have been explored [20], there are no “normal values” of the Hiding Heidi test. Thus, the results of this testing are qualitative. The value of this test is for evaluating for difficulty in perception of low contrast facial features at the child’s usual communication distances, since visual communication is critical during the first year of life. Thus, this test provides valuable information for planning future interventions and helps teachers and therapists understand the child’s requirements. We did not find a significant difference in the occurrence of visual impairment between preterm versus full term infants, or infants with or without structural eye abnormalities, microcephaly, risk for developmental delay, other neurodevelopmental abnormalities, or confirmed ZIKV infection. Among those with visual impairment, 68% did not have microcephaly, structural ocular abnormalities, or neurodevelopmental abnormalities. We theorize that visual impairment in these infants is likely due to cortical visual impairment based on the findings that the ZIKV directly targets and damages human cortical neural progenitor cells, with specificity for the eye and brain [21,22,23,24]. We feel that, similar to the wide spectrum of disease seen in those affected by ZIKV, there is likely a wide spectrum of damage to these neural progenitor cells, and while there may not be structurally obvious changes, there may be subclinical damage to the eye and the visual cortex that affects vision. This is supported by the fact that of the 14 infants with confirmed ZIKV infection in our cohort, only 1 (7.1%) had microcephaly. 

This report has limitations. One limitation is that a majority (154/242, 63%) of the total pregnancies from USVI in USZPIR did not attend the ZHB. Additionally, there is limited data on the infants who did not attend; thus, we could not determine if our results are generalizable to this population. The ZHB was performed in the aftermath of Hurricanes Irma and Maria, which had a significant impact on the USVI and may have limited attendance at the ZHB due to logistical difficulties (e.g., inability to contact, transportation, no longer living in the USVI) or socioeconomic strain. Moreover, natural disasters have been associated with reduced fetal growth, mental health issues in the post-natal period in both mothers and children, increased likelihood of acute illness, and decreased height and weight development in children [25,26,27,28]. The effect these natural disasters may have had on the development of these children and how they may have independently influenced the findings of this report is unknown [16]. Furthermore, there is no control or comparison group, and it is unclear if the findings of this report are more or less common than in the general population in the USVI. Comparisons made between our findings and previously published reports are limited by differences in subject inclusion criteria (e.g., CZS vs. possible ZIKV infection), age at time of evaluation, and how visual impairment was evaluated/defined. By defining visual impairment similarly to previous studies [5,6], we could make some direct comparisons. Another limitation of our study was that during the ZHB, children tended to have their ophthalmologic examination after extensive developmental pediatric, neurologic, and audiologic examinations. Thus, visual function testing may have been affected by fatigue and disinterest and would likely have overestimated visual impairment. Additionally, there was limited neuroimaging among our cohort; thus, it is possible that undetected brain abnormalities (e.g., mild brain findings like calcifications) could account for visual impairment found in our cohort, but we were unable to explore associations between the presence or absence of abnormal findings on neuroimaging and visual impairment. Another limitation is that our report describes findings at one point in time. There have been reports of manifestations of ZIKV infection that are not initially noted at birth, e.g., postnatal microcephaly in children born with normal head circumference [29]. Findings on ophthalmologic testing can also change over time, for example, change in visual acuity/function assessment can vary between examinations due to subject mood/cooperation, the presence of delayed visual maturation (i.e., abnormal visual function testing in a child with otherwise normal ocular and neurologic examination which by definition will resolve over time and visual function will normalize), and some ophthalmic manifestations (e.g., retinal findings) can evolve or change over time. One study noted that among children infected with ZIKV, development of visual acuity may be slowed even in the absence of microcephaly [11]. Thus, repeat examinations would help reveal the true prevalence and incidence of ophthalmic manifestations and visual impairment. 

It is important to continue expanding our knowledge of the ocular and visual effects of antenatal ZIKV infection during pregnancy. While there have been multiple reports describing the ophthalmic and visual findings in children severely affected by antenatal ZIKV infection [5,6,7,8], less has been reported on infants with the milder spectrum of congenital ZIKV infection [11,12]. We found that despite a low prevalence of structural ocular disease and microcephaly in our cohort, about a quarter of children had visual impairment. Furthermore, many of the children with visual impairment did not have a structural eye abnormality, microcephaly, or developmental abnormality. Careful monitoring, evaluation, and follow-up of children with congenital ZIKV infection is essential in early identification and appropriate referral for health services. As many of the methods used to evaluate for visual impairment in this study were qualitative in nature, it will be important to use more quantitative tests of visual function (e.g., visual acuity testing by optotypes) as this cohort of children becomes older and is able to perform these tests. This report further supports current recommendations that ophthalmological testing be performed in all children with suspicion for antenatal ZIKV infection, even in those with a normal ocular structure and neurologic examination [30]. 

## Figures and Tables

**Table 1:** Expected visual milestones based on age [17,18].

Adjusted Age	Milestone
8 weeks	Recognizes human face
3 months	Social smile and regards hands
5–6 months	Goal directed reach, moves to reach, hands midline
7–10 months	Recognizes facial expressions
12–18 month	Putting objects into and out of a container, reaches for dangling object
18–24 months	Scribbling

**Table 2:** Baseline demographic and clinical information ^a^.

Characteristics of Children Included in This Report	
**Gender**	
Male, n (%)	48 (59.3)
**Gestational age (weeks); n = 81**	
Mean (standard deviation (SD))	37.6 (2.9)
Median (min, max)	38.4 (27.6, 41.3)
**Preterm (Gestational age < 37 weeks) n (%)**	21 (25.9)
**Adjusted age at examination during ZHB (months); n = 81**	
Mean (SD)	9.1 (4.7)
Median (min, max)	8.1 (0.9, 21.9)
**Birth weight (grams); n = 67**	
Mean (SD)	2965 (693)
Median (min, max)	3120 (755–3960)
**Clinical Findings, n (%)**	
**Confirmed Zika virus infection in mother and/or infant ^b^**	14 (17.3)
**Microcephaly at birth**	5 (6.2)
**Developmental abnormalities ^c^**	19 (23.5)
**At risk for developmental delays (areas) ^d^**	18 (22.2)
Fine motor	8 (9.9)
Communication	7 (8.6)
Gross motor	6 (7.4)
Problem solving	4 (4.9)
Social-emotional	3 (3.7)
Personal-social	3 (3.7)
**Other neurodevelopmental abnormalities ^e^**	5 (6.2)
Abnormal tone	4 (4.9)
Arthrogryposis	1 (1.2)
Movement abnormality	1 (1.2)
Seizures	1 (1.2)

^a^ Among 81 children meeting the United States Zika Pregnancy and Infant Registry (USZPIR) eligibility criteria who attended the Zika Health Brigade (ZHB) and charts could be located. USZPIR eligibility criteria included children born between 1 December 2015 and 31 March 2018 in the United States Virgin Islands, to mothers with confirmed or possible recent zika virus (ZIKV) infection as defined by (1) recent ZIKV infection detected by Zika ribonucleic acid nucleic acid amplification test (NAAT) on any maternal, placental, fetal, or infant specimen or (2) recent ZIKV or flavivirus infection detected by serologic tests of maternal, fetal, or infant specimen [14]. ^b^ Confirmed ZIKV infection was defined as either: (1) positive NAAT or (2) positive or equivocal Zika Immunoglobulin M testing and ZIKV plaque reduction neutralization test (PRNT) ≥ 10 and dengue PRNT < 10. ^c^ Developmental abnormalities were considered present if the child was either categorized during the ZHB as “at risk for developmental delay” or found to have “other neurodevelopmental abnormalities”. Groups are not mutually exclusive. ^d^ Based on results of screening using the Ages and Stages Questionnaire −3. ^e^ Based on physical examination by a pediatric neurologist.

**Table 3:** Proportion of children with visual impairment, overall and by gestational age and other clinical characteristics ^a^.

	Overall ^b^	Gestational Age <37 Weeks	Structural Eye Abnormalities	Microcephaly	At Risk for Developmental Delay ^c^	Other Neuro-Developmental Abnormalities ^d^	Confirmed ZIKV Infection ^e^	Normal Eye, Neurologic, and Developmental Exam
	**n = 78**	**n = 21**	**n = 2**	**n = 5**	**n = 18**	**n = 5**	**n = 14**	**n = 58**
	**n (%)**	**n (%)**	**n (%)**	**n (%)**	**n (%)**	**n (%)**	**n (%)**	**n (%)**
**Visual Impairment**	**19/72** **(26.4)**	**6/19** **(31.6)**	**0/2** ** (0.0)**	**1/5 ** **(20.0)**	**5/16 ** **(31.3)**	**2/4 ** **(50.0)**	**5/13** **(38.5)**	**13/51** **(25.5)**
Hiding Heidi contrast testing	18/75 (24.0)	6/19 (31.6)	0/2 (0.0)	1/5 (20.0)	5/16 (31.3)	2/4 (50.0)	5/13 (38.5)	12/54 (22.2)
Shift of gaze	6/77 (7.8)	1/20 (5.0)	0/2 (0.0)	1/5 (20.0)	2/17 (11.8)	2/5 (40.0)	1/14 (7.1)	3/55 (5.5)
Visual fields	1/73 (1.4)	0/1 (0.0)	0/2 (0.0)	1/5 (20.0)	1/16 (6.3)	1/4 (25.0)	1/12 (8.3)	0/53 (0.0)
Accommodative reflex	1/71 (1.4)	0/18 (0.0)	0/2 (0.0)	1/5 (20.0)	1/15 (6.7)	1/4 (25.0)	1/13 (7.7)	0/52 (0.0)
Visual milestone	2/78 (2.6)	0/20 (0.0)	0/2 (0.0)	1/5 (20.0)	1/17 (5.9)	1/5 (20.0)	1/14 (7.1)	1/56 (1.8)

^a^ Among 81 children meeting the United States Zika Pregnancy and Infant Registry (USZPIR) eligibility criteria who attended the Zika Health Brigade and charts could be located. USZPIR eligibility criteria included children born between 1 December 2015 and 31 March 2018 in the United States Virgin Islands, to mothers with confirmed or possible recent zika virus (ZIKV) infection as defined by (1) recent ZIKV infection detected by Zika ribonucleic acid nucleic acid amplification test (NAAT) on any maternal, placental, fetal, or infant specimen or (2) recent ZIKV or flavivirus infection detected by serologic tests of maternal, fetal, or infant specimen [14]. ^b^ Of the 81 children, 3 children were unable to be tested. ^c^ At risk for developmental delay defined as delay in fine motor, communication, gross motor, problem solving, personal-social, and/or social-emotional domains based on results of screening using the Ages and Stages Questionnaire −3. ^d^ Other neurodevelopmental abnormalities included abnormal tone and/or movement, seizures, and arthrogryposis based on physical examination by a pediatric neurologist. ^e^ Confirmed ZIKV infection was defined as either: (1) positive NAAT or 2) positive or equivocal Zika Immunoglobulin M testing and ZIKV plaque reduction neutralization test (PRNT) ≥ 10 and dengue PRNT < 10.

**Table 4:** Comparison of the proportion of children with visual impairment by gestational age and other clinical characteristics ^a^.

	Visual Impairment n (%)	*p*-Value
**Full-term versus preterm**		
Preterm (gestational age <37 week)	6/19 (31.6)	0.56
Full-term (gestational age ≥37 weeks)	13/53 (24.5)	
**Structural eye abnormalities**		
With	0/2 (0.0)	1.0
Without	19/70 (27.1)	
**Microcephaly**		
With	1/5 (20.0)	1.0
Without	18/67 (26.9)	
**At risk for developmental delay ^b^**		
With	5/16 (31.3)	0.75
Without	14/56 (25.0)	
**Other neurodevelopmental abnormalities ^c^**		
With	2/4 (50.0)	0.28
Without	17/68 (25.0)	
**Confirmed ZIKV infection ^d^**		
With	5/13 (38.5)	0.31
Without	14/59 (23.7)	

^a^ Among 81 children meeting the United States Zika Pregnancy and Infant Registry (USZPIR) eligibility criteria who attended the Zika Health Brigade and charts could be located. USZPIR eligibility criteria included children born between 1 December 2015 and 31 March 2018 in the United States Virgin Islands, to mothers with confirmed or possible recent zika virus (ZIKV) infection as defined by (1) recent ZIKV infection detected by Zika ribonucleic acid nucleic acid amplification test (NAAT) on any maternal, placental, fetal, or infant specimen or (2) recent ZIKV or flavivirus infection detected by serologic tests of maternal, fetal, or infant specimen [14]. ^b^ At risk for developmental delay defined as delay in fine motor, communication, gross motor, problem solving, personal-social, and/or social-emotional domains based on results of screening using the Ages and Stages Questionnaire −3. ^c^ Other neurodevelopmental abnormalities included abnormal tone and/or movement, seizures, and arthrogryposis based on physical examination by a pediatric neurologist. ^d^ Confirmed ZIKV infection was defined as either: (1) positive NAAT or 2) positive or equivocal Zika Immunoglobulin M testing and ZIKV plaque reduction neutralization test (PRNT) ≥ 10 and dengue PRNT < 10.

**Table 5:** Comparison of ocular findings and visual assessment among infants/children with suspected or confirmed antenatal zika virus (ZIKV) infection previously reported and in this report.

	Zin et al. [8]	Vercosa et al. [7]	Ventura et al. [6]	Ventura et al. [5]	Portnoi Baran et al. [11]	Lima et al. [12]	Current Report
Year published	2018	2017	2017	2018	2019	2020	
Subjects	Infants with suspected ZIKV infection ^a^	Infants with microcephaly due to presumed congenital zika syndrome	Infants with neurologic or neuro-radiologic abnormalities with laboratory confirmed ZIKV infection ^b^	Infants born with laboratory confirmed ZIKV infection ^b^	Children exposed to or infected by ZIKV during gestation ^c^	Children exposed to or infected by ZIKV during gestation with at least 2 eye examinations ^c^	Infants/children with suspected ZIKV infection ^d^
Location	Brazil	Brazil	Brazil	Brazil	Brazil	Brazil	USVI
n	173	70	32	119	47	33	81
Age (months)	3–6 (range)	3.7 (mean, n = 25) ^e^	5.7 (mean)	8.5 (mean)	6.5 (mean)	NR	9.1 (mean)
**All findings listed below are by subject unless otherwise noted**							
**Structural Eye Abnormality**	26% (45/173)	26% (18/70)	44% (14/32)	46% (104/227) of eyes	2.1% (1/47)	3.0% (1/33)	3% (2/81)
Anterior segment abnormality	2% (4/173)	0% (0/70)	0% (0/32)	NR	NR	NR	1% (1/81)
Posterior segment abnormality	26% (45/173)	26% (18/70)	44% (14/32)	NR	2.1% (1/47)	3.0% (1/33)	1% (1/81)
Retinal abnormality	19% (33/173)	26% (18/70)	28% of eyes (18/64)	32% (74/234) of eyes	2.1% (1/47)	3.0% (1/33)	0% (0/81)
Optic nerve abnormality	22% (38/173)	14% (10/70)	17% of eyes (11/64)	27% (63/235) of eyes	NR	NR	1% (1/81)
**Microcephaly**	36% (62/173)	100% (70/70)	100% (32/32)	89% (100/113)	8.5% (4/47)	12.1% (4/33)	6% (5/81)
**Visual impairment**	30% (52/173) ^e^	100% (11/11) ^f^	100% (32/32) ^g^	90% (204/227) of eyes ^h^	10.6% (5/47) ^f^	3.0% (1/33) ^f^	26% (19/72) ^i^
Among those with microcephaly	71% (44/62)	100% (11/11)	100% (32/32)	NR	50.0% (2/4)	25.0% (1/4)	20% (1/5)
Among those with abnormal eye structure	84% (38/45)	100% (8/8)	100% (14/14)	96% (100/104) of eyes	100.0% (1/1)	100.0% (1/1)	0% (0/2)
Among those with normal eye structure	11% (14/128)	100% (3/3)	100% (18/18)	85% (104/123) of eyes	8.7% (4/46)	0.0% (0/32)	27% (19/70)
Among those with normal eye structure, neurologically normal, and no microcephaly or nystagmus	0% (0/87)	not applicable	not applicable	NR	7.0% (3/43) among those without microcephaly and/or structural ophthalmic abnormalities	0% (0/29) among those without microcephaly and/or structural ophthalmic abnormalities	26% (13/51)
**Type of visual acuity testing**	Fix & Follow	Teller acuity card	Teller acuity card	Teller acuity card	Teller acuity card	Teller acuity card	NR
**Abnormalities of the following:**							
**Visual acuity testing**	30% (52/173)	100% (11/11)	73% (22/30) of subjects 78% (47/60) of eyes	90% (107/119) of subjects 90% (210/233) of eyes	10.6% (5/47)	3.0% (1/33)	NR
**Visual function testing**	NR	NR	71% (22/31)	NR	NR	NR	27% (19/72)
Accommodation	NR	NR	36% (5/14)	NR	NR	NR	1% (1/71)
Hiding Heidi 5% contrast testing	NR	NR	65% (20/31)	81% (87/107)	NR	NR	24% (18/75)
Shift of gaze	NR	NR	42% (13/31)	NR	NR	NR	8% (6/77)
Visual field test	NR	NR	NR	45% (41/91)	NR	NR	1% (1/73)
**Visual developmental milestone**	NR	NR	97% (30/31)	93% (100/108)	NR	NR	3% (2/78)
**Strabismus**	14% (24/173)	14% (10/70)	75% (24/32)	80% (95/119)	NR	NR	1% (1/78)
**Nystagmus**	16% (27/173)	9% (6/70)	28% (9/32)	45% (54/119)	NR	NR	0% (0/80)

NR, not reported; USVI, United States Virgin Islands. ^a^ Based on positive reverse transcription polymerase chain reaction (RT-PCR) testing during pregnancy or infancy, prenatal ultrasound suspicious for ZIKV, or born with clinical manifestations of congenital ZIKV infection. ^b^ Based on the antibody-capture enzyme-linked immunosorbent assay method on cerebrospinal fluid. ^c^ Based on positive quantitative reverse transcription PCR (RT-qPCR) from urine and/or blood samples from mothers during pregnancy or children with positive result within 10 days of birth. Inconclusive RT-qPCR results were confirmed with ZIKV IgG and IgM serology. ^d^ Based on recent ZIKV infection detected by Zika ribonucleic acid nucleic acid test on any maternal, placental, fetal, or infant specimen or recent ZIKV or flavivirus infection detected by serologic tests of maternal, fetal, or infant specimen. ^e^ For 25 children with ocular abnormalities. ^f^ Extrapolated based on abnormal visual acuity testing, the term “visual impairment” not defined in report. ^g^ Based on abnormal Teller Acuity Card vision testing and/or abnormal visual function (Hiding Heidi contrast test, shift of gaze, light perception, response to the human face) or visual developmental milestone testing. ^h^ Based on abnormal Teller Acuity Card vision testing and/or abnormal visual function (Hiding Heidi contrast test, shift of gaze, visual field test, accommodation, light perception, response to the human face) or visual developmental milestone testing. ^i^ Based on abnormal visual function (Hiding Heidi contrast test, shift of gaze, visual field test, and accommodation) or visual developmental milestone testing.

## Data Availability

The data presented in this study are available on request from the corresponding author. The data are not publicly available due to patient privacy.
